# Analysis of a Potentially Suitable Habitat for *Solanum aculeatissimum* in Southwest China Under Climate Change Scenarios

**DOI:** 10.3390/plants14131979

**Published:** 2025-06-28

**Authors:** Shengyue Sun, Zhongjian Deng

**Affiliations:** 1 College of Forestry, Southwest Forestry University, Kunming 650224, China; 2 Yunnan Institute of Biodiversity, Kunming 650224, China

**Keywords:** *Solanum aculeatissimum*, MaxEnt, alien plants, potential geographical distribution

## Abstract

*Solanum aculeatissimum* is a herbaceous to semi-woody perennial plant native to the Brazilian ecosystem. It has naturalized extensively in southwestern China, posing significant threats to local biodiversity. This study systematically screened and integrated 100 distribution records from authoritative databases, including the Chinese Virtual Plant Specimen Database, the Global Biodiversity Information Facility, and Chinese Natural Museums. Additionally, 23 environmental variables were incorporated, comprising 19 bioclimatic factors from the World Climate Dataset, 3 topographic indicators, and the Human Footprint Index. The objectives of this research are as follows: (1) to simulate the plant’s current and future distribution (2050s/2070s) under CMIP6 scenarios (SSP1-2.6, SSP2-4.5, and SSP5-8.5); (2) to quantify changes in the distribution range; and (3) to determine the migration trajectory using MaxEnt 3.4.4 software. The findings reveal that human pressure (contributing 79.7%) and isothermality (bioclimatic factor 3: 10.1%) are the primary driving forces shaping its distribution. The core suitable habitats are predominantly concentrated in the provinces of Yunnan, Guizhou, and Sichuan. By 2070, the distribution center shifts northeastward to Qujing City. Under the SSP5-8.5 scenario, the invasion front extends into southern Tibet, while retreat occurs in the lowlands of Honghe Prefecture. This study underscores the synergistic effects of socioeconomic development pathways and bioclimatic thresholds on invasive species’ biogeographical patterns, providing a robust predictive framework for adaptive management strategies.

## 1. Introduction

With the rapid advancement of international exchanges and global trade, an increasing number of alien species have been intentionally or unintentionally introduced into extensive regions across the world. When these species extend beyond their natural distribution ranges and experience exponential growth, thereby causing detrimental impacts on local ecosystems or economies, they are categorized as alien species [1]. When a naturalized alien species establishes itself in a new habitat, issues typically result, such as a reduction in local biodiversity through ecological niche competition and other mechanisms. Biological invasions are considered one of the most significant challenges in the 21st century. Crucially, climate is the primary factor constraining *Solanum aculeatissimum’s* distribution [2,3,4]. In the context of substantial global climate change, comprehending the spatial distribution of biological populations and their interactions with the environment is essential for elucidating biodiversity patterns and developing effective conservation strategies.

*Solanum aculeatissimum*, which is native to Brazil and Paraguay, is a herbaceous or subshrub species belonging to the Solanaceae family and the *Solanum* genus. It has extensively dispersed across tropical regions in Asia and Africa, where it commonly thrives in roadside thickets, abandoned lands, grassy slopes, and sparse woodlands. Notably, all parts of the plant contain toxic alkaloids, and accidental ingestion by humans or livestock may induce poisoning symptoms. Prior research has primarily focused on the development of its medicinal potential. The plant contains significant amounts of solanine and solasonine, which serve as critical raw materials for the synthesis of steroid hormones and are key precursors for the artificial production of steroid drugs and sex hormones [5,6]. This species exhibits remarkable adaptability in natural environments, which is attributed to its seed dormancy mechanism, cold tolerance, and resilience to adverse environmental conditions, enabling it to maintain viability for extended periods under harsh circumstances. Furthermore, *Solanum aculeatissimum* demonstrates strong resistance to diseases and a high degree of adaptability to varying light conditions, allowing it to thrive even under 95% shading [7,8].

Given its alien potential and adaptability, understanding the factors governing its distribution in specific regions is critical. This is particularly relevant for China, where *Solanum aculeatissimum* is primarily distributed in Yunnan Province, with sporadic records also reported in the Guangxi Zhuang Autonomous Region [9,10]. Although prior studies have elucidated the biological characteristics, utilization value, and geographical distribution of *Solanum aculeatissimum*, a significant knowledge gap persists, and research on the influence of climatic factors in southwestern China on its distribution remains limited. Renowned for its diverse topography and significant altitudinal gradients, southwestern China offers favorable natural conditions for the growth and dispersal of *Solanum aculeatissimum,* heightening concerns about its further spread. Therefore, the primary objectives of this study are as follows: (1) identify the key climatic factors limiting the distribution of *Solanum aculeatissimum* in southwestern China and (2) predict its current and future potential distribution areas under climate change scenarios. We hypothesize that specific temperature and precipitation variables are the dominant climatic constraints on its distribution in this region.

With the advancement of information technology, numerous models have been developed to predict the potential distribution areas of species, including CLIMEX, GARP, DIVA-GIS, BIOCLIM, and MaxEnt. Among these, the MaxEnt model has gained widespread application in ecology and biogeography for species distribution modeling. It is particularly noted for its ability to maintain high prediction accuracy and precision, even when the distribution data are limited [11,12]. MaxEnt transforms the environmental factors of the study area into raster data and performs statistical analysis based on known distribution points, thereby estimating the probability of species distribution in each raster cell and the relationship between environmental variable values and distribution probabilities [13,14,15]. Consequently, we employ the MaxEnt model in the context of predicting naturalized alien plant species, as it can assist researchers in identifying environmental conditions conducive to their growth, thereby enabling the prediction of their potential distribution areas.

## 2. Results

### 2.1. Key Influence Factors

**Figure 1:**
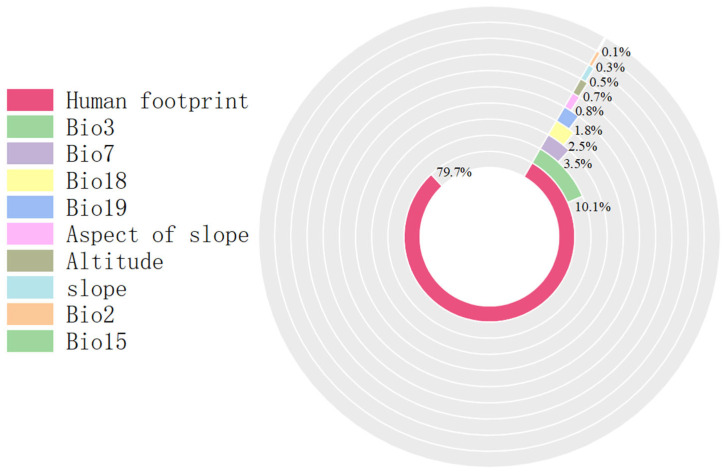
Proportion chart for each influencing factor.

**Figure 2:**
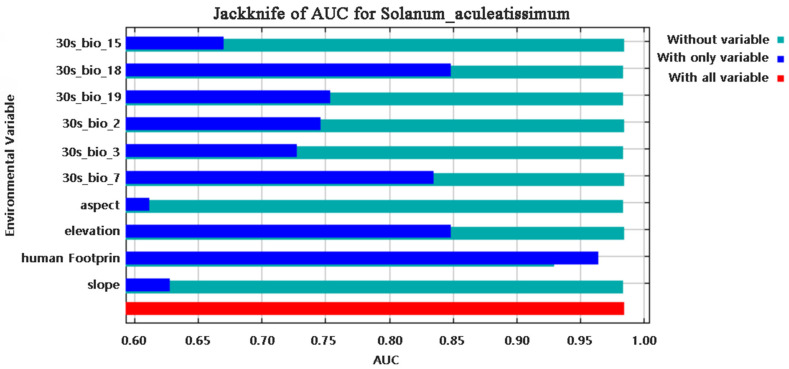
Results of the jackknife method for the influencing factors of *Solanum aculeatissimum*.

**Figure 3:**
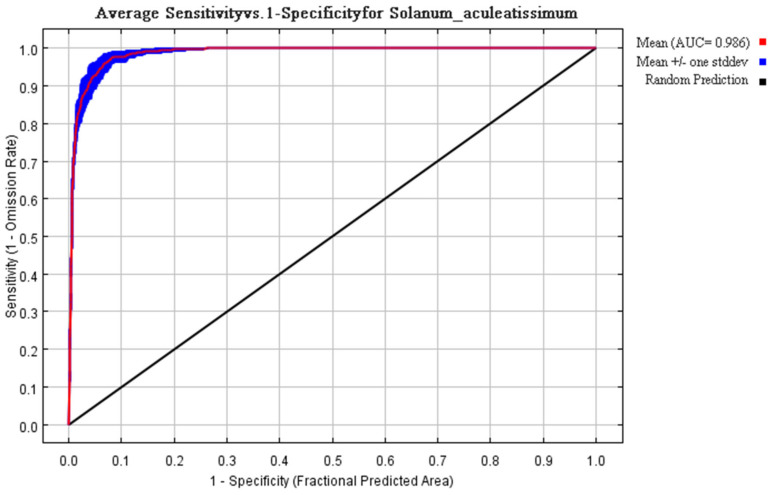
During the verification process, the classification accuracy of the model was assessed using metrics obtained from repeated training iterations. Across 10 computational trials, the algorithm achieved a mean AUC value of 0.986 ± 0.003, demonstrating its robust performance and reliability.

Multivariate analysis identified four principal determinants governing the potential distribution of *Solanum aculeatissimum xishuangbannaensis* in southwestern China: thermal regimes (Bio2 [mean diurnal range], Bio3 [isothermality], Bio7 [temperature annual range]), precipitation variability (Bio15 [precipitation seasonality], Bio18 [warmest quarter precipitation], Bio19 [coldest quarter precipitation]), topographic gradients (elevation, slope, aspect), and anthropogenic pressure (human footprint). Permutation importance metrics ranked anthropogenic factors as the dominant driver (79.7%), noticeably exceeding bioclimatic variables (Figure 1). Jackknife sensitivity testing confirmed these three variables as the primary determinants of regularized training gain, demonstrating disproportionate influence compared to other predictors (Figure 2). The AUC-ROC index was employed to assess the habitat suitability of *Solanum aculeatissimum*. The findings revealed that the model exhibited exceptional performance in predicting the species’ habitat suitability. During the iterative process of the model, the mean AUC value was 0.986 with a standard deviation of ±0.003, indicating the high consistency and reliability of the analysis results. These outcomes were based on 10 computational experiments, further underscoring the model’s accuracy in evaluating the ecological niche of *Solanum aculeatissimum* (Figure 3).

### 2.2. Contemporary Climate-Driven Habitat Suitability for Solanum aculeatissimum

**Figure 4:**
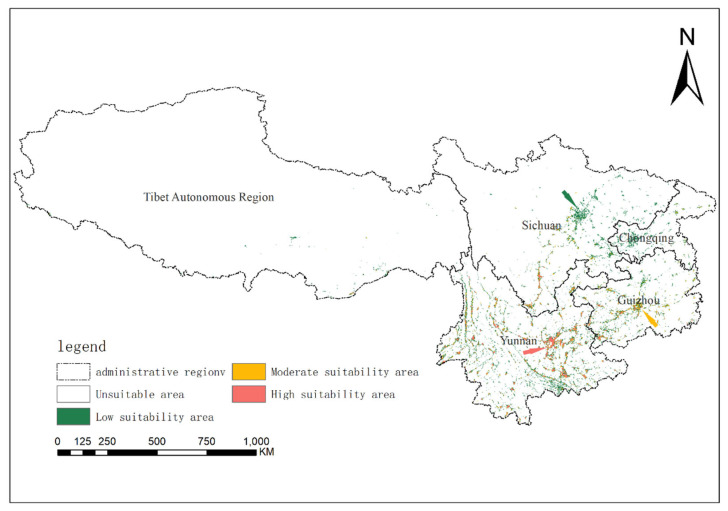
The potential geographical distributions of *Solanum aculeatissimum* in Yunnan Province, Sichuan Province, Guizhou Province, Tibet Autonomous Region, and Chongqing City of China under the current climate.

Habitat suitability stratification across the study area revealed pronounced spatial heterogeneity. Non-suitable habitats spanned approximately 21.1 × 10^4^ km^2^ (~97.12% of total area), predominantly occupying the Tibetan Autonomous Region. Low-suitability zones (~4.35 × 10^4^ km^2^, ~2.01%) clustered in Sichuan Province and Chongqing Municipality. Moderate-suitability areas (~1.00 × 10^4^ km^2^, ~0.46%) concentrated in central Guizhou Province, while high-suitability nuclei (~0.88 × 10^4^ km^2^, ~0.41%) formed two distinct clusters: (1) central and eastern Yunnan, including Kunming City, Honghe Hani and Yi Autonomous Prefecture, and Wenshan Zhuang and Miao Autonomous Prefecture; and (2) western Guizhou, notably Liupanshui City (Figure 4).

### 2.3. Climate Change Impacts on Habitat Suitability for Solanum aculeatissimum

**Figure 5:**
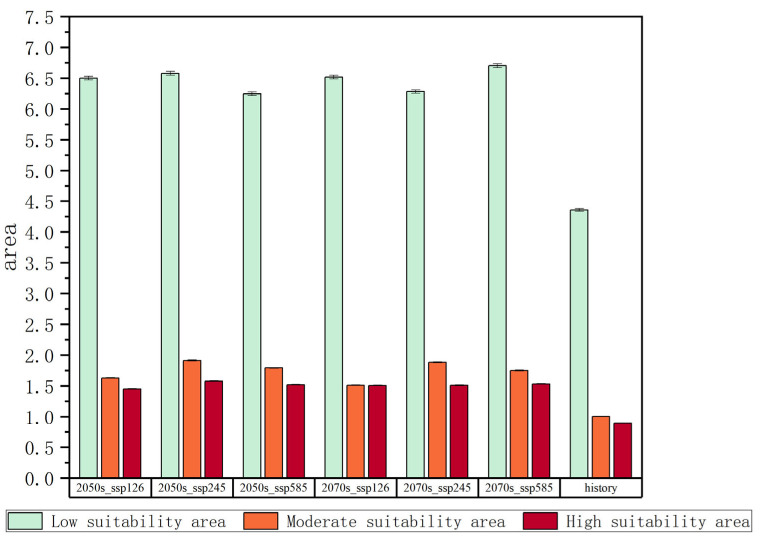
Changes in the areas of different suitable habitats.

Three CMIP6 scenarios (SSP1-2.6, SSP2-4.5, and SSP5-8.5) were analyzed to assess future climate impacts on the potential distribution of *Solanum aculeatissimum* in the mid-century (2050s) and late-century (2070s) periods. High-suitability habitats remained persistently concentrated in central–southern Yunnan Province, encompassing Kunming City and the Honghe–Wenshan ecoregion. Moderate-suitability and low-suitability zones dominated Sichuan and Guizhou provinces, with a pronounced expansion of moderate-to-high-suitability areas observed across all scenarios (Figure 5). Under SSP1-2.6, western Sichuan exhibited progressive suitability declines, transitioning from moderate-suitability to low-suitability classifications between 2050 and 2070.

To systematically investigate the spatiotemporal dynamics of *Solanum aculeatissimum* potential distribution patterns, this study evaluated projected habitat suitability shifts under three climate change scenarios (SSP1-2.6, SSP2-4.5, and SSP5-8.5) for the mid-21st-century (2050s) and late-century (2070s) periods. Through integrated multidimensional modeling incorporating environmental covariates and biogeographic constraints, coupled with rigorous statistical validation, we quantified the spatial extent and distribution characteristics of the high-suitability/moderate-suitability zones relative to the total study domain. The analysis revealed distinct temporal trajectories in habitat suitability class distributions across different emission scenarios, with particularly notable variations observed in optimal growth areas.

**Figure 6:**
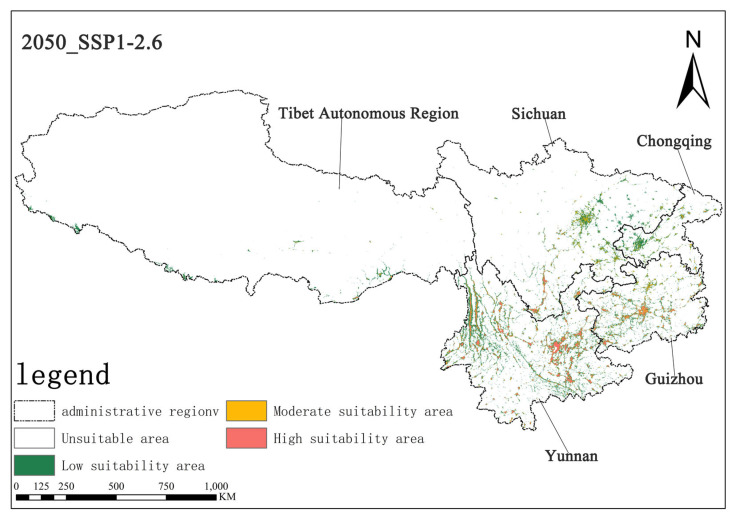
Illustration of potential distribution areas by the 2050s under the SSP1-2.6 scenario.

**Figure 7:**
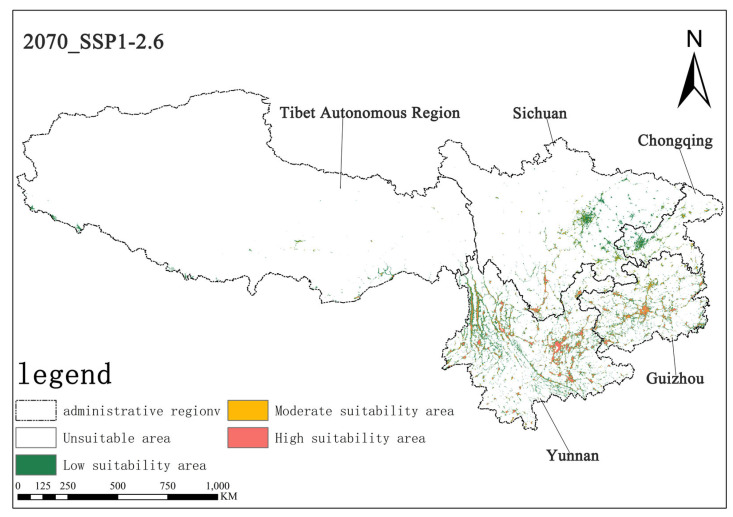
Illustration of potential distribution areas by the 2070s under the SSP1-2.6 scenario.

Under the SSP1-2.6 scenario, the high-suitability area for *Solanum aculeatissimum* in the 2050s is projected to cover approximately 1.44 × 10^4^ km^2^ (0.67% of the total study area), while the medium-suitability area spans 1.62 × 10^4^ km^2^ (0.75%). By the 2070s, the high-suitability area is projected to expand to 1.50 × 10^4^ km^2^ (0.69%), whereas the medium-suitability area will decline slightly to 1.51 × 10^4^ km^2^ (0.70%). These results suggest a modest increase in high-suitability habitats, along with a marginal contraction in medium-suitability habitats, with minimal net change in overall suitable distributions (Figure 6 and Figure 7).

**Figure 8:**
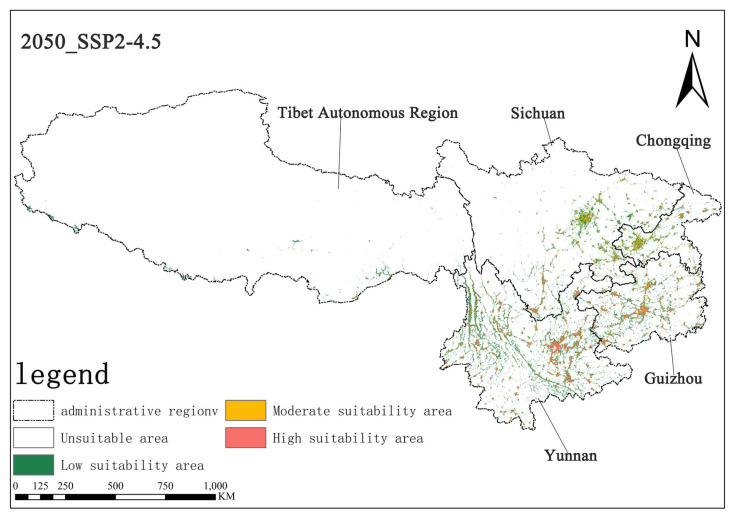
Illustration of potential distribution areas by the 2050s under the SSP2-4.5 scenario.

**Figure 9:**
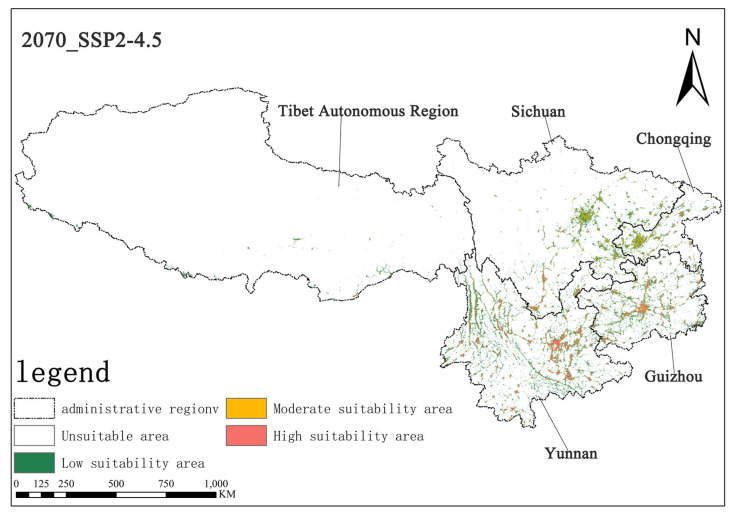
Illustration of potential distribution areas by the 2070s under the SSP2-4.5 scenario.

Under the SSP2-4.5 scenario, in the 2050s, the area of highly suitable habitats for *Solanum aculeatissimum* is projected to be approximately 1.57 × 10^4^ square kilometers, accounting for 0.73% of the total study area, while the moderately suitable habitat area will be approximately 1.91 × 10^4^ square kilometers, making up 0.88%. By the 2070s, the area of the highly suitable habitat is projected to decrease to approximately 1.50 × 10^4^ square kilometers, with the proportion dropping to 0.70%, and the moderately suitable habitat area is also reduced to about 1.88 × 10^4^ square kilometers, representing 0.87%. It can be observed that, in this scenario, from the 2050s to the 2070s, the areas of both the highly suitable and moderately suitable habitats for *Solanum aculeatissimum* will shrink, but proportional changes will be relatively stable (Figure 8 and Figure 9).

**Figure 10:**
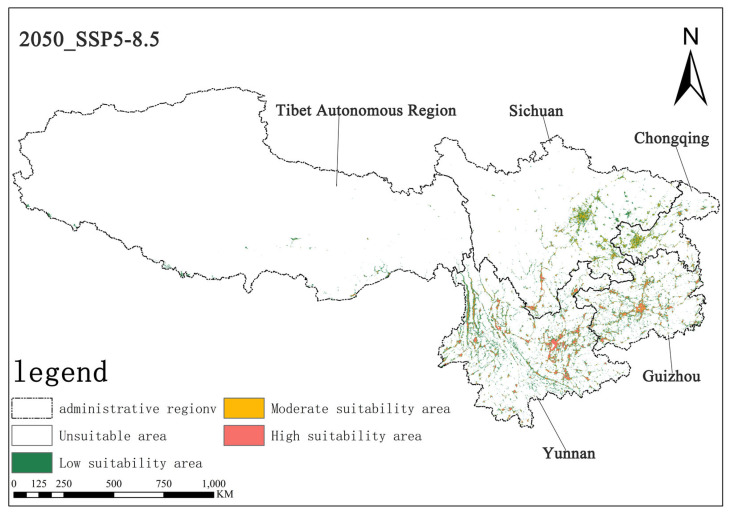
Illustration of potential distribution areas by the 2050s under the SSP5-8.5 scenario.

**Figure 11:**
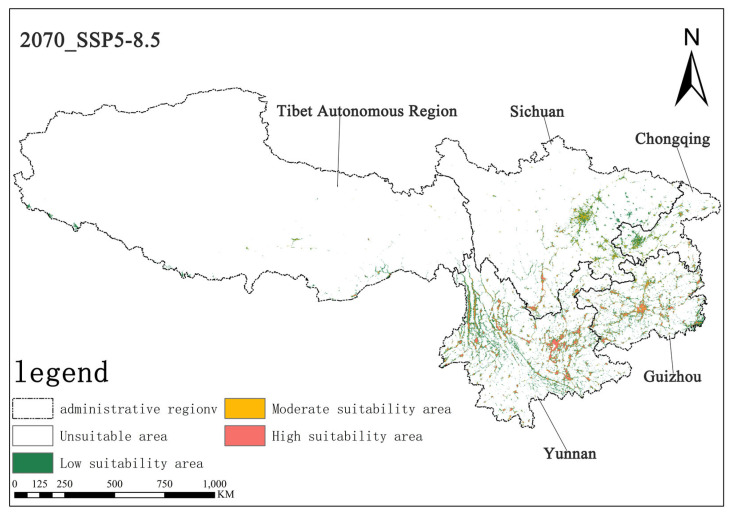
Illustration of potential distribution areas by the 2070s under the SSP5-8.5 scenario.

Under the SSP5-8.5 scenario, the high-suitability area for *Solanum aculeatissimum* was projected at 1.15 × 10^4^ km^2^ (0.70% of total area) for the 2050s, noticeably increasing to 1.53 × 10^4^ km^2^ (0.71%) relative to the 2070s. Concurrently, the medium-suitability areas decreased slightly from 1.78 × 10^4^ km^2^ (0.82%) to 1.75 × 10^4^ km^2^ (0.81%) over the same period. These results demonstrate a notable expansion in high-suitability habitats accompanied by a marginal contraction in medium-suitability areas (Figure 10 and Figure 11).

The three SSP scenarios revealed considerable divergence in projected habitat suitability for *Solanum aculeatissimum*. SSP1-2.6 exhibited limited variation in high-suitability and medium-suitability areas, whereas SSP2-4.5 exhibited a net decline in the total suitable habitat area. In contrast, SSP5-8.5 demonstrated the pronounced expansion of high-suitability zones (33% increase from 2050s to 2070s), along with minimal contraction in medium-suitability areas (−1.7%), highlighting scenario-dependent ecological responses.

### 2.4. Contraction and Dilation Zone

This study analyzed the changing trends of the stable, contraction, and expansion areas of *Solanum aculeatissimum* under different Shared Socioeconomic Pathways (SSP1-2.6, SSP2-4.5, and SSP5-8.5) until the 2070s, aiming to reveal the influence mechanisms of different development scenarios on spatial pattern evolution.

**Figure 12:**
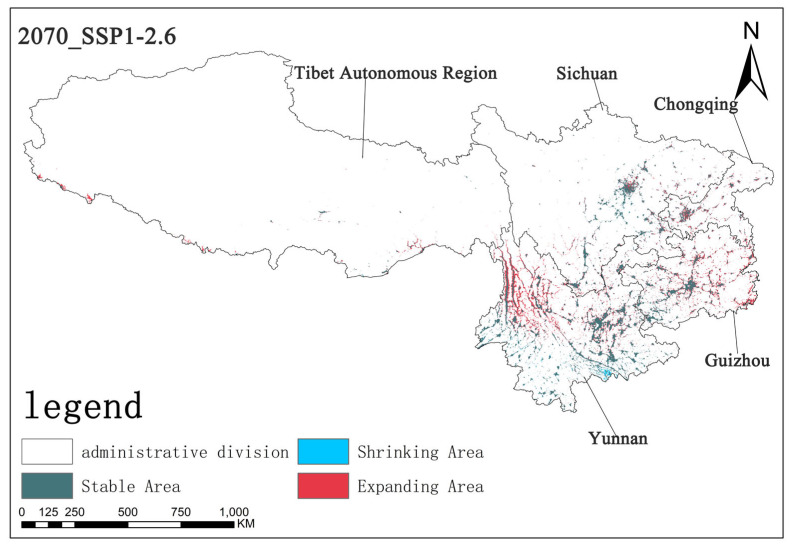
The diagram illustrating contraction and expansion under the SSP1-2.6 scenario up to the 2070s.

Under the SSP1-2.6 scenario, by the 2070s, the stable area is approximately 5.67 × 10^4^ km^2^, the shrinking area is about 0.32 × 10^4^ km^2^, and the expanding area is approximately 3.56 × 10^4^ km^2^. In this scenario, the stable area is relatively large, the shrinking area is small, and the expanding area is of a certain scale, indicating that the overall pattern of *Solanum aculeatissimum* is relatively stable under this scenario, but there is still a certain degree of regional expansion (Figure 12).

**Figure 13:**
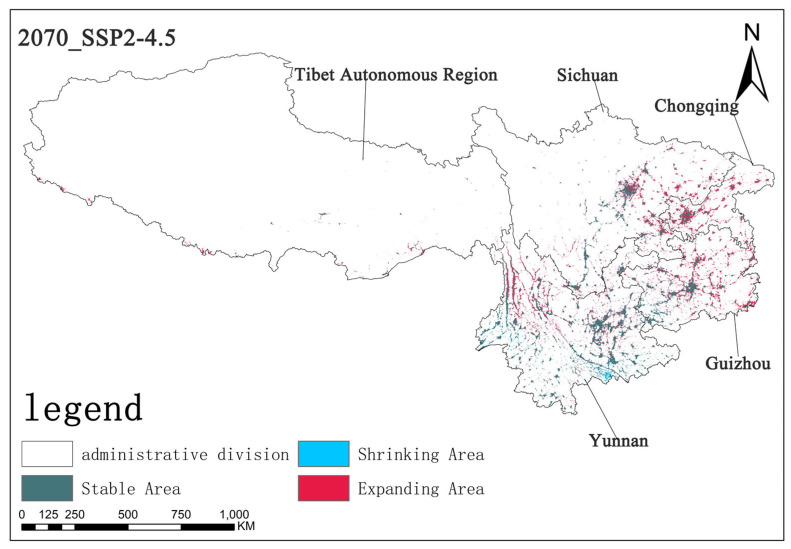
The diagram illustrating contraction and expansion under the SSP2-4.5 scenario up to the 2070s.

Under the SSP2-4.5 scenario, by the 2070s, the stable area is approximately 5.62 × 10^4^ km^2^, the shrinking area is about 0.38 × 10^4^ km^2^, and the expanding area is approximately 3.72 × 10^4^ km^2^. Compared with the SSP1-2.6 scenario, the stable area decreased slightly, the shrinking area increased, and the expanding area increased. This indicates that, under this scenario, the stability of *Solanum aculeatissimum*’s survival slightly declined, and the expansion trend relatively strengthened, but the overall change is not significant (Figure 13).

**Figure 14:**
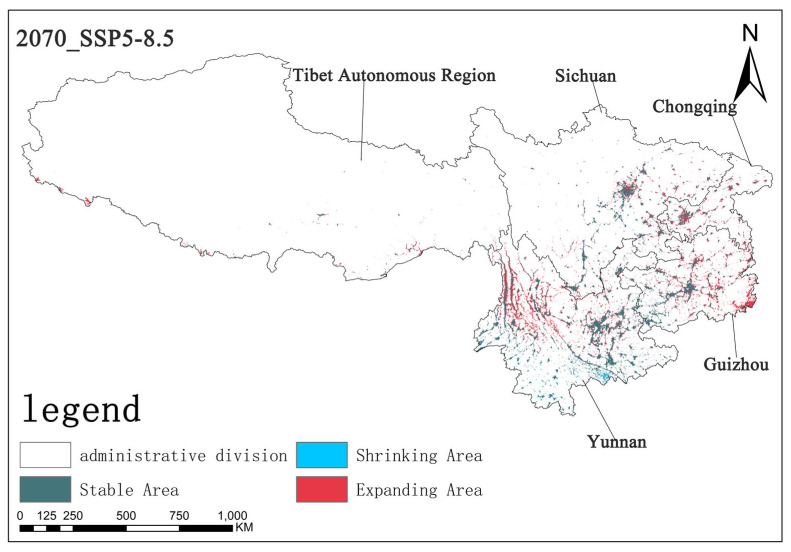
The diagram illustrating contraction and expansion under the SSP5-8.5 scenario up to the 2070s.

Under the SSP5-8.5 scenario, by the 2070s, the stable area is approximately 5.65 × 10^4^ km^2^, the contraction area is about 0.34 × 10^4^ km^2^, and the expansion area is approximately 4.01 × 10^4^ km^2^. Compared with the previous two scenarios, the expansion area changed noticeably in this scenario, while the contraction area did not noticeably change. The stable area is at an intermediate level. This indicates that the expansion trend of *Solanum aculeatissimum* is the most obvious in this scenario, and spatial pattern changes are more active (Figure 14).

Based on the analysis of the three scenarios, different shared socioeconomic pathways have exerted varying influences on the regional changes of the research subject. The expansion trend is most prominent under the SSP5-8.5 scenario, indicating that there might be more favorable factors for the expansion of *Solanum aculeatissimum* in this scenario, such as environmental changes and socioeconomic development models. Through the analysis model, it was observed that the main expansion areas are located in the northwestern part of Yunnan Province, the eastern part of Guizhou Province, and some areas of Chongqing Municipality. Notably, there are also obvious expansion areas in the southern border region of the Tibet Autonomous Region, such as the southern part of Shannan City, the southern part of Xigaze City, and the southern part of Ngari Prefecture. Meanwhile, there are significant contraction areas in the Honghe Hani and Yi Autonomous Prefecture in the southern part of Yunnan Province (Figure 11, Figure 12 and Figure 13).

### 2.5. Average Center-Point Shift

The current average distribution center of *Solanum aculeatissimum* is situated within Kunming City, Yunnan Province. Under the SSP1-2.6 scenario, by the 2050s, the average distribution center will shift northeastward and be located within Qujing City, Yunnan Province; by the 2070s, it will move southeastward but remain confined to Qujing City, Yunnan Province. Under the SSP2-4.5 scenario, by the 2050s, the average distribution center will also shift northeastward into Qujing City, Yunnan Province; however, by the 2070s, it will not shift southeastward as in the SSP1-2.6 scenario but instead continue moving northwestward while still remaining within Qujing City, Yunnan Province. Under the SSP5-8.5 scenario, by the 2050s, the distribution center will shift northeastward and enter Qujing City, Yunnan Province; by the 2070s, it will shift southwestward but remain within Qujing City, Yunnan Province.

**Figure 15:**
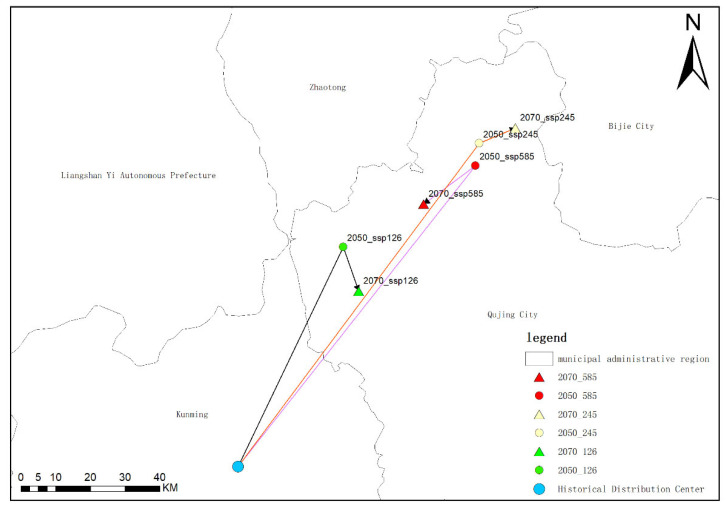
Diagrams of the average central points of the distribution of *Solanum aculeatissimum* Jacq. in Yunnan Province, Sichuan Province, Guizhou Province, Chongqing Municipality, and Tibet Autonomous Region in the 2070s under the scenarios of SSP1-2.6, SSP2-4.5, and SSP5-8.5.

Through an analysis of the shift in the average center of species distribution, it can be observed that the overall shift direction of *Solanum aculeatissimum* in Yunnan Province, Sichuan Province, Guizhou Province, Chongqing Municipality, and the Tibet Autonomous Region is predominantly towards the northeast. Under the SSP1-2.6, SSP2-4.5, and SSP5-8.5 scenarios, the distribution center will shift to the area around Qujing City, Yunnan Province, by the 2070s. However, it is noteworthy that under the SSP1-2.6 and SSP5-8.5 scenarios, there will be a reverse shift phenomenon towards the southwest by the 2070s. In particular, in the SSP5-8.5 scenario during the 2070s, the distribution center exhibits a pronounced trend that returns towards the southwest (Figure 15).

## 3. Discussion

### 3.1. Key Environmental Drivers of Solanum aculeatissimum Potential Distribution

This study employed the MaxEnt model to systematically analyze various factors influencing the potential distribution area of *Solanum aculeatissimum*, quantifying their contribution rates, importance rankings, and the results of the jackknife test. Consequently, the primary factors affecting the potential distribution area of *Solanum aculeatissimum* were identified. This analysis not only elucidates the intricate relationship between the distribution of *Solanum aculeatissimum* and environmental factors but also offers a critical foundation for further exploring its ecological adaptability and spread mechanisms.

The model’s analysis reveals that the environmental factors influencing the potential distribution area of *Solanum aculeatissimum* exhibit diverse and complex characteristics. The varying contribution rates of these factors reflect the specific environmental requirements and adaptation strategies of *Solanum aculeatissimum.* Identifying the primary influencing factors is essential for understanding the ecological traits of *Solanum aculeatissimum*. These factors not only define its current distribution range under existing environmental conditions but may also shape its future expansion trends and invasion potential. From an ecosystem perspective, the introduction of alien species may disrupt the balance of native ecosystems and threaten local biodiversity. The expansion or contraction of *Solanum aculeatissimum’s* distribution range could influence the structure and function of local ecosystems. By investigating the relationship between its potential distribution area and environmental factors, we can more effectively evaluate the potential impacts of *Solanum aculeatissimum’s* spread, implement preventive and intervention measures proactively, and safeguard the stability and biodiversity of ecosystems.

#### 3.1.1. Anthropogenic Factors

The research findings indicate that in Yunnan, Sichuan, and Guizhou Provinces, Chongqing Municipality, and the Tibet Autonomous Region of China, the human footprint is the predominant factor influencing the potential distribution area of *Solanum aculeatissimum*, accounting for 79.7%. The dispersal of non-native species driven by human activities represents a significant aspect of global environmental change [16]. With the growing globalization of trade, non-native plant species are continuously being introduced to various parts of the world via trade goods, representing a significant factor driven by human activities between countries. Concurrently, advancements in transportation have enhanced population mobility, further exacerbating the spread of non-native plants. Since the launch of China’s “Belt and Road Initiative” in 2013, southwestern China has emerged as a key communication hub for Southeast Asia, resulting in a corresponding increase in the risk of naturalized species. Among these regions, Yunnan Province, which is the focus of this study, has been the most noticeably impacted. Research indicates that, in addition to natural factors, human activities also play a critical role in determining the extent of naturalized plant distribution. Economic and demographic variables serve as indicators of the intensity of human activities and incorporate factors that directly influence invasion processes, such as reproductive pressure, eutrophication, and the degree of human-induced disturbance [17,18]. In the context of plant invasion prevention and control, intentional introductions by humans have emerged as one of the key driving factors for the invasion of alien plant species. The deliberate cultivation of ornamental plants represents the primary pathway for the introduction of such species [19]. The findings of this study indicate that the areas with high suitability for *Solanum aculeatissimum* are predominantly concentrated in densely populated provincial capital cities, including Kunming, Guiyang, Chengdu, and Chongqing. While human activities noticeably influence the dispersal of naturalized plants, controlling their spread solely through restrictions on population movement poses an immense challenge. Consequently, exploring feasible and effective control strategies for naturalized plants while ensuring economic development remains a pressing research priority that requires immediate attention.

#### 3.1.2. Climatic Drivers

Another critical factor influencing the potential distribution area of *Solanum aculeatissimum* in southwestern China, identified while conducting prediction research for this species, was Bio3 (Isothermality), which had a contribution rate of 10.1%. Climate serves as a critical selective force that shapes plant traits, ranging from physiological characteristics to life history features and defense mechanisms [20,21,22,23,24,25,26]. The findings of this study are in agreement with those reported by Zhang et al. [27], indicating that the primary bioclimatic and environmental factors influencing the spatial geographical distribution of *Solanum aculeatissimum* include precipitation during the hottest season and the annual temperature range. Under historical climatic conditions, the potential distribution area of *Solanum aculeatissimum* was predominantly concentrated in regions with higher temperatures, and the number of aggregation areas in low-altitude regions was noticeably greater than that in high-altitude regions. Given that *Solanum aculeatissimum* is native to tropical regions and exhibits limited adaptability to cold, high-altitude areas, its naturalization in high-altitude and cold regions was relatively low under historical climatic conditions.

### 3.2. Contraction–Expansion Range Dynamics

In predicting the contraction and expansion zones of *Solanum aculeatissimum*, this study selected three Shared Socioeconomic Pathways (SSP1_2.6, SSP2_4.5, and SSP5_8.5) for the 2070s and compared the changes in distribution areas under different pathways. Our research revealed that *Solanum aculeatissimum* exhibited not only an expansion trend within the study area but also a relatively distinct contraction zone in the southern part of Yunnan Province.

For the expansion zone, the human footprint, as the most substantial contributing factor, has been elaborated upon in the preceding section. With global warming, naturalized plant species are progressively encroaching from low-latitude, low-altitude, and high-temperature regions into high-latitude, high-altitude, and low-temperature zones. Although nations have contributed greater commitments and established more ambitious goals to address climate change, current policies remain inadequate in limiting the rise in global temperatures to approximately 2.7 degrees Celsius above pre-industrial levels by the end of this century [28,29,30,31]. *Solanum aculeatissimum* is native to tropical regions and exhibits high sensitivity to environmental temperatures. With climate change, temperatures in low-temperature areas at high latitudes and altitudes have been progressively increasing, thereby creating favorable conditions for the colonization of this species within these regions. In this study, irrespective of the socioeconomic scenario, *Solanum aculeatissimum* exhibits a clear expansion trend into low-temperature areas at high latitudes and altitudes. This trend is particularly pronounced in the Tibet Autonomous Region and the northwestern part of Yunnan Province. However, this study also revealed that under the influence of the three Shared Socioeconomic Pathways (SSP1_2.6, SSP2_4.5, and SSP5_8.5), there were significant contraction zones in certain regions, predominantly concentrated in the southern part of Yunnan Province, including Pu’er City, Xishuangbanna Dai Autonomous Prefecture, and Honghe Hani and Yi Autonomous Prefecture. With climate warming, the migration of species toward high latitudes and altitudes represents a dominant trend; however, warm-edge contraction or local extinction should also be taken into consideration. Under the combined effects of climate warming and interspecies interactions, the distribution range of species is likely to expand in cold-edge areas (high latitudes and high altitudes) while contracting in warm-edge areas (low latitudes and low altitudes) [32]. This phenomenon is consistently observed in plants across all analyses and is typically evident in animals as well. Overall, the extinction rate of animals is noticeably higher than that of plants, with this disparity being more pronounced when comparing temperate rather than tropical species. Due to the inherent characteristics of the global climate system, the impact of climate change is spatially pervasive on a global scale. Climate systems in both high latitudes/altitudes and low latitudes/altitudes are undergoing dynamic changes, which are often accompanied by the formation of compounded natural disaster chains, including extreme precipitation events, abnormal high temperatures, droughts, and biological disasters. These climate stressors substantially influence the native distribution area of *Solanum aculeatissimum* by altering habitat suitability. According to the ecological threshold theory, even minor climate fluctuations may trigger critical thresholds in species distribution, resulting in an exponential increase in the extinction risk of local populations.

From the perspective of community ecology, the theory of niche differentiation elucidates the coexistence mechanism of species within ecosystems. When the climatic parameters of the original habitat deviate from the ecological amplitude tolerated by a species, alien plant groups that are better-adapted to new climatic conditions will occupy ecological niche spaces through diffusion processes. In climate contraction zones, due to dominant factors—such as temperature and precipitation exceeding the tolerance range of *Solanum aculeatissimum*—its key physiological indicators, including photosynthetic efficiency and reproductive success rate, will noticeably decline. According to resource competition theory, in ecosystems with limited resources, this adaptive disadvantage will result in its gradual replacement in interspecific competition, ultimately resulting in regional extinction events. The warm-edge contraction phenomenon observed in this study’s results is highly consistent with the conclusion drawn by LENOIR J (2010) [32], suggesting that when conducting prevention and control efforts for *Solanum aculeatissimum* invasion, attention should not only be paid to its diffusion characteristics but also vigilance maintained regarding the potential niches created by warm-edge contraction, which may serve as settlement opportunities for other naturalized plants.

## 4. Materials and Methods

### 4.1. Data Sources

The distribution point data for *Solanum aculeatissimum* were primarily obtained from the China Virtual Herbarium (www.cvh.ac.cn) and the China Nature Museum (www.cfh.ac.cn). By reviewing the specimen images available on these platforms, the locations of occurrence of *Solanum aculeatissimum* in Yunnan Province, Guizhou Province, Sichuan Province, Chongqing Municipality, and the Tibet Autonomous Region were identified, and their geographical coordinates were determined using GIS (10.8) software. Additionally, the distribution coordinates of *Solanum aculeatissimum* in the aforementioned regions were statistically analyzed based on data retrieved from the Global Biodiversity Information Facility (GBIF, www.gbif.org). In total, 27 data points were collected from the China Virtual Herbarium, 9 data points from the China Nature Museum, and 346 data points from GBIF.

However, it is important to highlight that during the subsequent practical operational phase, the initial dataset of 382 records underwent a rigorous cleaning process, ultimately resulting in the utilization of 98 points. Specifically, duplicate records (within an approximate 1 km radius) were removed, as were the points located outside of the study area due to statistical inaccuracies or errors originating from an excessively distant historical period. Consequently, this yielded 98 spatially unique and environmentally relevant presence points suitable for modeling purposes. To mitigate potential spatial biases arising from imbalanced sampling, background points were systematically sampled across the entire region, and spatial independence validation was employed to assess model performance.

Environmental variables: Environmental variable data were sourced from the WorldClim Database (https://www.worldclim.org), which provides historical (1970–2000) and projected climate data with a spatial resolution of 30 arcseconds (approximately 1 km × 1 km). Future climate data were derived from the BCC-CSM2-MR global climate model, a climate system model developed by the Beijing Climate Center (BCC) under the framework of the Sixth Coupled Model Intercomparison Project (CMIP6). The future climate data cover two periods—the 2050s (2041–2060) and the 2070s (2061–2080)—corresponding to three Shared Socioeconomic Pathways (SSPs): SSP1-2.6 (sustainable development), SSP2-4.5 (moderate development), and SSP5-8.5 (high-emission development) [33]. Both historical and future datasets include 19 bioclimatic variables (Bio1–Bio19). Additionally, elevation, slope, and aspect data were obtained from the WorldClim 2.1 elevation dataset at a spatial resolution of 30 arcseconds. Human footprint data were provided by the Urban Environment Monitoring Team of the College of Land Science and Technology, China Agricultural University (www.x-mol.com/groups/li_xuecao (accessed on 21 December 2024)). Eight variables reflecting different aspects of human pressure were utilized: built environment, population density, night-time lights, farmland, pastures, roads, railways, and navigable waterways. Following the methods of Sanderson and Venter et al., the annual dynamic data of the global terrestrial human footprint from 2000 to 2020 were generated. Mapping accuracy was assessed using Venter’s visual interpretation samples (a total of 3460). The results indicated that the R^2^ of this human footprint map was 0.62, which is higher than that of the previous human footprint map (R^2^ = 0.50). Additionally, it demonstrated strong consistency with the datasets developed by Venter, Kennedy, and Williams et al., achieving correlations of 0.72, 0.66, and 0.93, respectively.

### 4.2. Data Processing

**Figure 16:**
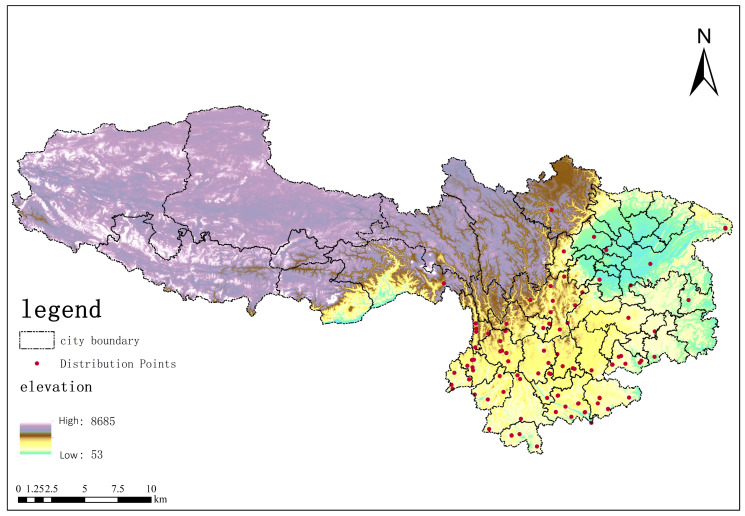
Khasi Thorn (*Solanum aculeatissimum*) distribution map in Yunnan Province, Sichuan Province, Guizhou Province, Chongqing Municipality, and Tibet Autonomous Region.

Distribution point data processing: Using the Environmental Niche Models Tools (ENMTools 5.26) software, the distribution points of *Solanum aculeatissimum* were systematically screened. ENMTools is a specialized software platform for analyzing ecological niche models, enabling the quantitative analysis of ecological niches, similarity measurements, and statistical testing, further facilitating interactions with the MaxEnt model [34,35,36]. ENMTools is capable of automatically adjusting the analysis of the raster size for environmental factors, removing redundant data within the same raster, and preventing overfitting during the prediction of species’ habitat suitability distribution using the MaxEnt model [37]. After screening, the original 382 distribution data points were reduced to 100 (as shown in Figure 16) and subsequently converted into CSV format for model operation.

**Figure 17:**
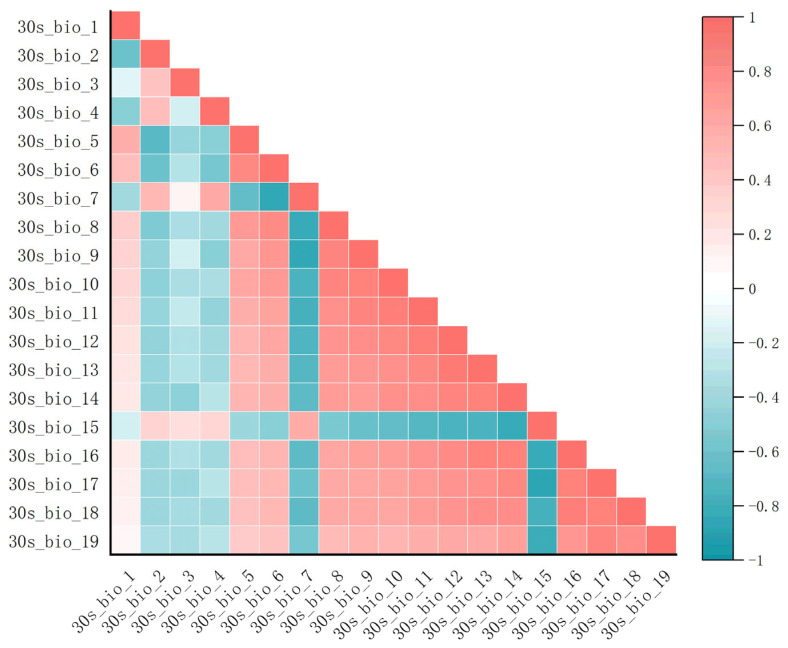
Climate factor correlation heatmap.

Climate factor data processing: Environmental variables serve as critical data for constructing ecological niche models. Utilizing an excessive number of environmental variables may result in collinearity issues, consequently diminishing the accuracy of the model [38,39]. Therefore, all influencing factors were initially input into MaxEnt for a preliminary run to filter out factors with a contribution rate of 0. Subsequently, using ENMTools software, a correlation analysis is performed on all candidate variables (as shown in Figure 17). When the correlation coefficient was |r| > 0.8, the variables were classified as highly correlated [40]. For highly correlated data pairs (with |r| > 0.8), during the initial maximum entropy calculation, we retained variables with higher contribution rates and excluded those with lower contribution rates to ensure model efficiency and reduce redundancy. Ultimately, after excluding the influencing factors with a contribution rate of 0 and those with a correlation coefficient of |r| > 0.8 and relatively low contribution rates, a total of 6 climate and environmental factors, 3 topographic factors, and 1 human footprint factor were retained for model construction (as presented in Table 1). The final set of variables comprises climate variable data for Bio2, Bio3, Bio7, Bio15, Bio18, and Bio19; topographic variable data for Elevation, Slope, and Exposure; and human footprint data for Human Footprint. For further details, we refer readers to Table 1. Subsequently, the selected factors were converted into asc format files for future utilization.

### 4.3. Calculation of Suitable Habitat Area

The MaxEnt model was employed to estimate the suitable habitat for *Solanum aculeatissimum*. Various regularization multipliers and feature class parameters were configured within the model. During model execution, 75% of the samples were allocated for training purposes, while the remaining 25% were utilized for model validation. The model was executed 10 times, with a maximum of 5000 iterations and 10,000 background points specified. The relative contribution of each environmental variable was assessed using the jackknife method [41,42]. The accuracy of the MaxEnt model was assessed using the Area Under the Curve (AUC) method of the Receiver Operating Characteristic (ROC) curve. Theoretically, an AUC value between 0.5 and 0.7 indicates poor performance, a value between 0.7 and 0.9 indicates average performance, and a value above 0.9 indicates good performance [43].

### 4.4. Suitability-Based Habitat Zoning

The values in the raster output of the MaxEnt model range from 0 to 1. Using ArcGIS 10.8 software, the model’s results were visualized. The raster was reclassified into four suitability levels using the reclassification method: 0–0.1 as non-suitable areas, 0.1–0.3 as low-suitability areas, 0.3–0.5 as medium-suitability areas, and 0.5–1 as high-suitability areas.

### 4.5. Definition of Contraction and Expansion Zones

The historical climate average data calculated using the MaxEnt model, as well as the 2070 scenario data for SSP1-2.6, SSP2-4.5, and SSP5-8.5, were imported into ArcGIS 10.8 software. First, the data were reclassified using the reclassification method. In this study, the ranges of suitability were defined as follows: 0–0.1 as non-suitable areas, 0.1–0.3 as low-suitability areas, 0.3–0.5 as medium-suitability areas, and 0.5–1 as high-suitability areas. Areas with values greater than 0.1 were extracted as suitable regions. Subsequently, the processed data files were converted to vector format and analyzed using the intersection analysis method within the overlay analysis toolkit. Finally, the resulting vector files were converted back to raster format to delineate the potential distribution and expansion areas of *Solanum aculeatissimum* under the influence of the SSP1-2.6, SSP2-4.5, and SSP5-8.5 climate scenarios relative to 2070. Based on the identified expansion trends, the distribution areas were categorized into three classes: stable areas, contraction areas, and expansion areas.

### 4.6. Average Center Point Shift

The historical climate average data calculated using the MaxEnt model, as well as the 2050 and 2070 scenario data under SSP1-2.6, SSP2-4.5, and SSP5-8.5, were imported into ArcGIS 10.8 for analysis. First, these data were reclassified into four suitability levels, and the ‘Value’ field was used to extract regions with values of 2, 3, or 4 via the attribute selection function. Next, the raster data were converted to vector format using the Raster to Polygon tool. Subsequently, the Mean Center tool in the Spatial Statistics Tools toolbox was employed to calculate the mean center points of the data of different periods. Finally, these mean center points were connected using lines to visualize the migration trajectories of the average center points of suitable areas influenced by the climate factors of SSP1-2.6, SSP2-4.5, and SSP5-8.5 in 2050 and 2070.

## 5. Conclusions

Based on the MaxEnt model, this study predicted a potential suitable habitat for *Solanum aculeatissimum* in southwestern China and simulated its future suitable habitat patterns under the SSP1-2.6, SSP2-4.5, and SSP5-8.5 scenarios for the 2050s and 2070s. Through multi-scenario analysis, the following core conclusions were drawn:

1. The human footprint and isothermality are the two most critical factors influencing the distribution of *Solanum aculeatissimum*. In contrast, geographical factors, such as altitude, aspect, and slope, have relatively limited effects on its distribution pattern;
2. Based on the simulation and prediction of future climate conditions, *Solanum aculeatissimum* is likely to expand into high-latitude and high-altitude low-temperature regions within the study area; simultaneously, suitable habitats in low-latitude and low-altitude areas are expected to exhibit a contracting trend;
3. Under future climate conditions, the centroid of *Solanum aculeatissimum’s* distribution in the study area generally demonstrates a northeastward migration trend; however, under specific climate scenarios, there may be partial southwestward retraction.

From an ecological perspective, the findings of this study elucidate the intricate relationship between the distribution of *Solanum aculeatissimum* and environmental factors, thereby enriching the research content in plant ecology concerning the distribution and ecological adaptability of naturalized species. Additionally, the results highlight the sensitivity of plants to climate change, demonstrating that even minor fluctuations in key climatic variables such as temperature and precipitation can noticeably alter the distribution range and growth status of plants. This underscores the necessity of paying greater attention to the interactions between plants and climate, as well as the impacts of climate change on ecosystems in the context of global climate change. Furthermore, this study empirically illustrates the growing importance and urgency of conducting in-depth research on plant response mechanisms under global climate change. Future studies could focus more extensively on the physiological and ecological response mechanisms of *Solanum aculeatissimum* to varying climate conditions—such as changes in photosynthesis, respiration, and reproductive capacity under temperature and precipitation variations—and how these changes influence its distribution and spread.

## Figures and Tables

**Table 1:** Selected factor table.

Variables	Variable Descriptions
Bio2	Mean diurnal range
Bio3	Isothermality
Bio7	Temperature annual range
Bio15	Precipitation seasonality
Bio18	Precipitation of warmest quarter
Bio19	Precipitation of coldest quarter
Elevation	Elevation of the study area
Slope	Elevation and gradient of the study area
Exposure	Aspect of the slope in the study area
Human Footprint	The intensity of human impact in the study area

## Data Availability

The data presented in this study are available upon request from the corresponding author.
